# Supplementary Motor Area Activity Differs in Parkinson's Disease with and without Freezing of Gait

**DOI:** 10.1155/2023/5033835

**Published:** 2023-09-04

**Authors:** J. Sebastian Marquez, Ronny P. Bartsch, Moritz Günther, S. M. Shafiul Hasan, Or Koren, Meir Plotnik, Ou Bai

**Affiliations:** ^1^Department of Electrical and Computer Engineering, Florida International University, Miami, Florida, USA; ^2^Department of Physics, Bar-Ilan University, Ramat Gan 5290002, Israel; ^3^Max Planck Institute for Meteorology, Hamburg, Germany; ^4^Center of Advanced Technologies in Rehabilitation, Sheba Medical Center, Ramat Gan, Israel; ^5^Department of Physiology and Pharmacology, School of Medicine, Tel Aviv University, Tel Aviv, Israel; ^6^Sagol School of Neuroscience, Tel Aviv University, Tel Aviv, Israel

## Abstract

The study aimed to investigate the neural changes that differentiate Parkinson's disease patients with freezing of gait and age-matched controls, using ambulatory electroencephalography event-related features. Compared to controls, definite freezers exhibited significantly less alpha desynchronization at the motor cortex about 300 ms before and after the start of overground walking and decreased low-beta desynchronization about 300 ms before and about 300 and 700 ms after walking onset. The late slope of motor potentials also differed in the sensory and motor areas between groups of controls, definite, and probable freezers. This difference was found both in preparation and during the execution of normal walking. The average frontal peak of motor potential was also found to be largely reduced in the definite freezers compared with the probable freezers and controls. These findings provide valuable insights into the underlying structures that are affected in patients with freezing of gait, which could be used to tailor drug development and personalize drug care for disease subtypes. In addition, the study's findings can help in the evaluation and validation of nonpharmacological therapies for patients with Parkinson's disease.

## 1. Introduction

Persons with Parkinson's disease (PD) demonstrate gait disturbances that usually worsen with disease progression. Freezing of gait (FoG) is an extremely debilitating one [1]. One of the hypotheses for explaining its underlying pathophysiology states that FoG is caused by a problem with the central drive and automaticity of movement [2].

It has also been hypothesized that the automaticity of movement mediated by the crosstalk of basal ganglia (BG) inputs from the motor, cognitive, and limbic cortical areas, which regularly complement each other in unaffected persons, is disrupted in PD with FoG. In addition, in the later stages of PD, as gait control becomes less automated, crosstalk between these competing regions worsens gait execution during dual-tasking [3]. To support this hypothesis, clear biomarkers are needed that are associated with active gait and the physiological response from neural activity in preparation for and during active gait.

Although EEG does not provide the high spatial resolution of other imaging modalities, its high temporal resolution and portability make it an ideal technique for investigating FoG during locomotor activities and quantifying related neural changes. Among the first who studied FoG using EEG were Shine et al. reporting alterations in particular brain waves during FoG [4–6]. Lower beta frequencies seem to be most affected by freezers during treadmill walking [7], and increased power in the high-beta band has been found for freezers during rest [8]. In contrast, interhemispheric EEG synchronization is increased across all frequency bands for PD patients [9], and EEG synchronization networks show the highest connectivity for those patients suffering from FoG [10]. While in our previous works [9–11], we have investigated EEG synchronization during regular walking episodes irrespective of the phases of the gait cycle, the present paper focuses on gait initiation (which is thought to be one of the triggers of FoG [12]). In addition, to improve the signal-to-noise ratio, event-related potentials were explored.

A feature of event-related potentials, the Bereitschaftspotential (BP), or “readiness potential” in German, is a fluctuation that appears over averaged time-locked EEG events, 1-2 seconds before limb movement and indicates movement preparation and readiness (see more in Supplementary Materials (available here)). The BP has several distinguishing components that precede voluntary movement, including late BP and the frontal peak of motor potential (fpMP). The late BP occurs after the initial rise in electrical activity and continues up to the point of movement initiation. It is thought to reflect the final stages of motor planning and preparation, including the selection of a specific motor plan and the suppression of alternative plans, and has been shown to originate over the primary motor cortex and represent motor activation. In this context, patients with Parkinson's disease have been shown to have a reduced late BP, which may reflect deficits in motor planning and preparation. The fpMP is represented by the highest amplitude 50 ms after onset and reflects sensory feedback after motor control. In addition to these BP features, time-frequency analysis is another method for evaluating neural patterns. A frequency-specific power increase or decrease in neural activity is known as an event-related synchronization (ERS) and event-related desynchronization (ERD), respectively.

The main aim of the present study is to investigate whether noninvasively obtained EEG biomarkers may be used to differentiate between healthy controls (HC), persons with PD with definite FoG, and those diagnosed with FoG but who did not exhibit any FoG events during the experimental trials, referred to, in this report, as “probable” freezers.

## 2. Methods

Experimental trials were conducted at the center of advanced technologies in rehabilitation (CATR) at Sheba Medical Center (Tel HaShomer, Israel). The experimental protocol was approved by the Sheba Medical Center Institutional Review Board. All participants signed informed consent prior to entering the study. Data collected in these trials were used, in part, for previous publications [9–11], where we investigated interhemispheric EEG synchronization and EEG brain networks but not event-related potentials. For more details on the experimental design and demographics, see the Supplementary Materials (available here).

### 2.1. Data Acquisition and Feature Extraction

Participants wore a portable EEG-EMG system (MicroMED®, Mogliano Veneto, Italy), and EEG was sampled at 2048 Hz employing a 32-channel EEG montage (“10–20 system”) consisting of Fz, Cz, Pz, Oz, Fp1, Fp2, F7, F8, F3, F4, FT9, FT10, FC5, FC6, T3, T4, C3, C4, CP5, CP6, TP9, TP10, T5, T6, P3, P4, O1, O2, PO9, and PO10 referenced at C7 and grounded on the left mastoid process. For gait cueing, surface EMG activity was recorded from four channels sampled at 2048 Hz, located at the tibialis anterior and gastrocnemius muscles of each leg. In addition to these objective measures, non-sensor-based metrics in the form of clinical scales were also recorded. These include the Unified Parkinson's Disease Rating Scale (UPDRS) and the Montreal Cognitive Assessment (MoCA). All trials were recorded using video synchronized with the physiological signal recording system for post hoc annotations. Across overground walking types, each freezing episode was annotated from video footage, and then individual epochs with freezing episodes 2.5 seconds before or after onset were excluded from the analysis. To explore both temporal and time-frequency features, EEG data were processed for both MRCP and ERD/S features. In brief, the EEG data were time-locked to electromyography spikes to mark the initialization of the first step, which indicated the onset of movement. Individual epochs were then averaged across trials to reduce noise and extract the underlying brain activity related to that event. Even though all participants with PD were considered as suffering from the FoG symptom, not all exhibited FoG episodes during at least one gait task. Thus, in a post hoc analysis, the PD group was split further into definite and probable, the former included five patients who exhibited FoG events during at least one of the gait-related tasks and the latter included five patients who did not exhibit FoG events during any of the gait-related tasks (but nevertheless usually experience FoG). A total of 31 ± 2 trials were averaged for each group in the MRCP analysis.

For in-depth details on these methods, refer to the Supplementary Materials (available here).

### 2.2. Statistical Analysis

Following baseline removal and frequency binning for time-frequency decomposition, each subject's trials were averaged to result in a single event for each, resulting in 5 samples for the HC group, 5 for the probable group, and 5 samples for the definite group. Because of the small patient sample size, the normal distribution could not be assumed. Instead, we utilized nonparametric statistics, which helped with the multiple comparisons' problem. We generated a permutation distribution by partitioning groups and creating random draws and constructed the empirical null distribution using the maximum t-value for each iteration of each permuted time series. A *p* value of 0.05 indicated statistical significance in probing for the observed differences between group means in both tails of the distribution. In the analysis of power change oscillations, statistics were computed in frequency bins of 2 Hz. In the temporal domain of event-related potentials, statistics were computed at the component of interest. Early BP statistics were computed using the slope from −500 ms to onset, and the frontal peak of motor potential statistics was computed at the first peak after onset.

## 3. Results

### 3.1. Probable Freezers vs. HC

**Figure 1:**
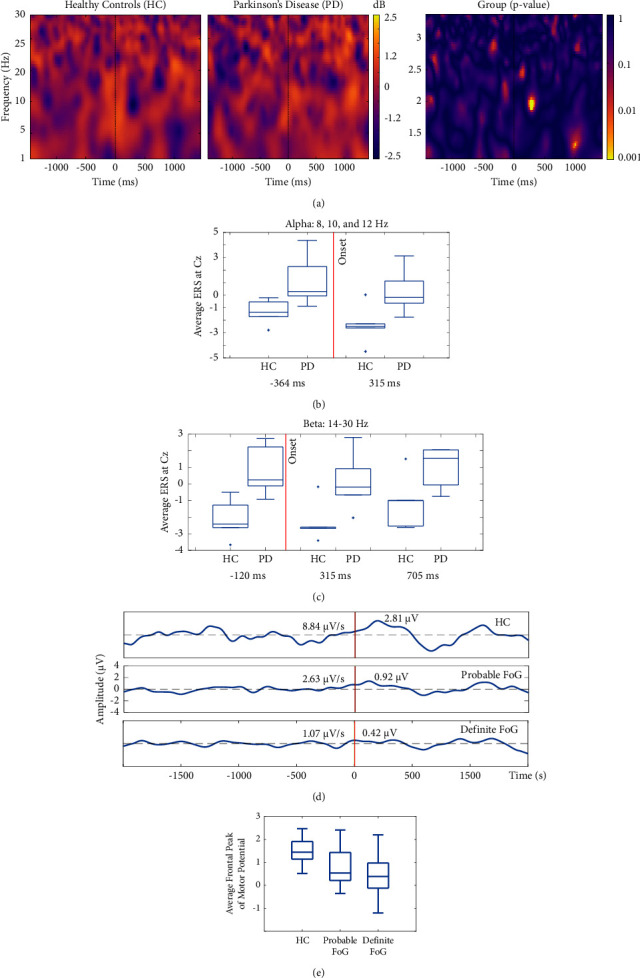
(a) Time-frequency comparison. Each time-frequency decomposition plot is made up of the average activity for each group from the Cz channel, which is close to the primary motor cortex. The *x*-axis represents the time 1500 ms before and 1500 ms after movement onset. The *y*-axis represents each frequency bin. Dark colored spots indicate event-related desynchronizations, while light colored spots correspond to event-related synchronizations. The rightmost plot depicts the intertrial coherence, with light color representing periods of significance between group trials. The PD group shows significantly less alpha and low-beta desynchronization at the motor cortex about 315 ms after the start of normal walking as compared to HC. Randomization testing supports this finding with *p*=0.039 for the comparison of frequency bins starting at 6 Hz and going to 12 Hz, 315 ms after the start of normal walking. (b, c) In these plots, the magnitude of the event-related desynchronization is marked by a negative value on the *y*-axis. Each PD-HC pair shown constitutes a statistically significant difference in the canonical band group with respect to time, with the red vertical line indicating the onset of walking. (b) Event-related desynchronization in the alpha band is significantly decreased for the PD group at 364 ms before onset and at 315 ms after onset. (c) Likewise, event-related desynchronization at the beta band is significantly decreased for the PD group compared with the HC group, 364 ms before onset and 315 and 705 ms after onset. (d) Averaged (Cz-C3) movement-related cortical potentials (MRCP) between 5 subjects for each group. Both definite and probable groups show significantly less late BP and fpMP in preparation and during the execution of normal walking, compared with HC. (e) The average frontal peak of motor potential is most largely reduced in the definite compared with the probable group and HC group.

Time-frequency decomposition yielded no significant differences when comparing probable vs. definite freezers. Thus, we combined all participants with PD into one PD group for this analysis. As seen in Figure 1(a), time-frequency decomposition results show a relative decrease in both alpha and beta activities in the PD group compared with the HC. This relative decrease was observed on the Cz channel 315 ms after the start of normal walking. The Cz ERD from 364 ms before to 705 ms after the start of normal walking is summarized in the boxplots presented in Figures 1(b) and 1(c). Channel Cz showed significant differences between the HC and PD groups with *p*=0.039 and moderate effect sizes, with Cohen's d of 0.51.

### 3.2. HC vs. Definite Freezers vs. Probable Freezers

The average between C3 and Cz, which constitute the sensory and motor areas, respectively, showed significant differences between the late BP and the frontal peak of motor potential (fpMP) between the three groups. The amplitudes of the MRCPs for each group are shown in Figure 1(d). For MRCP, the analysis was limited only to BP2, which indicates activation of the PMC. The HC group showed a clear polarization of BP2 about 500 ms before the movement onset with a late BP of 8.84 *μ*V/s. This was attenuated in both probable freezers at 2.63 *μ*V/s and definite freezers at 1.07 *μ*V/s. After onset, the fpMP was also attenuated in both probable freezers at 0.92 *μ*V 132 ms after onset and definite freezers at 0.42 *μ*V 56 ms after onset compared with HC at 2.81 *μ*V 80 ms after onset, with the largest attenuation seen for the definite freezers.

## 4. Discussion

### 4.1. Summary of Findings

In our study, the group of persons with PD showed lower alpha and beta ERD compared with HC during overground walking (Figures 1(a)–1(c)). Furthermore, MRCP features showed that within the PD group, those with definite FoG showed a decrease in the late BP slope leading to an overall lower late BP magnitude compared to those persons with PD probable FoG and HC (Figures 1(d) and 1(e)). This finding supports the notion that EEG features may be associated with FoG severity [8]. These MRCP features can serve as biomarkers to differentiate these FoG subtypes or severity, which may lead to better-targeted symptom management and improved personalized treatment.

### 4.2. Interpretation of Results

Herein, we interpret the present results considering the normal physiology of walking and previous findings on FoG. Note that the results and interpretation were made with respect to persons with Parkinson's disease who all have reported FoG symptoms, not a generic Parkinson's disease population.

**Figure 2:**
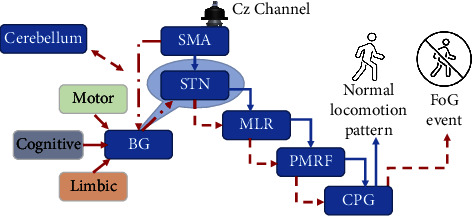
Modeling the neuronal processes potentially affecting cortical activity captured by the CZ electrode. Normal function is shown in blue arrows. The SMA starts the cueing for self-initiated actions, such as volitional walking, standing from a chair, or turning. This signal travels via the supraspinal locomotor network towards the mesencephalic locomotor region (MLR) through the hyperdirect pathway that connects the SMA and the subthalamic nucleus (STN). The STN acts as an intermediary between SMA and the MLR, and it is responsible for gating the SMA feedforward and cerebellar feedback from activating or inhibiting the MLR. The MLR then triggers the pontomedullary reticular formation (PMRF), which drives the central pattern generators (CPG) to produce the control patterns for locomotion [13]. This control loop is facilitated by dopamine; without it, an intention does not successfully convey movement. Disrupted function is shown in red arrows. It has been recently proposed that in PD, FoG may be caused by disrupted basal ganglia (BG)-SMA cues due to episodic crosstalk from the motor, cognitive, and limbic areas. This event-triggered crosstalk leads to gait initiation lock or FoG [14]. It is also plausible that impaired connectivity between the cortico-BG circuits and the cerebellum contribute to the appearance of FoG in PD [15] potentially reflecting neuronal activity deficits also in the cerebellum [16], which was implicated with complex gait conditions [17]. BG: basal ganglia, CPGs: central pattern generators, MLR: mesencephalic locomotor network, PMRF: pontomedullary reticular formation, SMA: supplementary motor area, SN: substantia nigra, STN: subthalamic nucleus, TH: thalamus, and VTA: ventral tegmental area.

Figure 2 shows schematically a relevant neuronal pathway involved in generating locomotion. Alteration of this pathway may lead to FoG, considering that the cortical activity over the SMA is reduced in PD before self-initiated movement because of the reduced neuronal input from the BG. In PD, the preparatory desynchronization (before movement initiation) is attenuated, followed by reduced synchronization after movement execution, compared with HC. One may argue that if our findings on preparatory desynchronization are indeed associated with FoG, they may be only considered relevant for “start hesitation” type of FoG (e.g., Schaafsma et al. [9]). We posit, however, that FoG events are generally triggered by the need to modify the gait pattern, as in the case of turning (i.e., “turning FoG”). These gait modifications require the initiation of motor programming to update locomotion function, which is impaired in persons with definite FoG, as reflected by the decreased alpha and beta desynchronization reported here in the context of gait initiation.

This study points to the lack of ERD in the PD group before and after the start of normal walking, potentially caused by affected movement programming, which is associated with reduced activity in the SMA. In addition, in the PD group, the ERS, which precedes ERD at the movement onset, may have been affected by the decreased ERD, resulting in an elongated, augmented ERS compared with HC. In an ankle dorsiflexion study, others also found decreased beta activity over Cz before movement onset [18].

Concerning MRCPs, previous studies have shown that the late BP is overactivated and is considered a compensation mechanism to achieve limb control without information handover from the basal ganglia to the SMA in PD [19]. With regards to FoG, Shoushtarian et al. reported on the relationship between stride length changes and MRCPs in PD patients with and without FoG [20]. In accordance with findings from our study, Karimi et al. showed that in ankle dorsiflexion, persons with PD and definite FoG have significantly lower early BP than healthy controls, and this difference was associated with the severity of freezing, suggesting an association between FoG subtype and severity. It is important to note that although overground gait and dorsiflexion represent different types of motion and evoke different neural patterns, freezing can manifest across different tasks, and subtypes based on these differences should continue to be evaluated.

Reduced late BP amplitude may also indicate less neuronal signal projection from BG to the SMA, leading to reduced gait readiness, caudally leading to FoG, or difficulty in self-initiated movement. On the other hand, higher late BP has been related to the compensatory mechanisms employed by PD to execute motor control [12]. The reduction in late BP slope has been matched with a reduction in stride length, which has also been shown to indicate FoG events [13]. This could be caused by the difference in the tasks administered, as this study explored changes during active gait, not index finger flexion. The additional cognitive load associated with gait execution, which in normal function is cued by the supraspinal locomotor network, becomes disrupted due to inhibition of the GPi/SNr pathway. This pathway breakdown causes engagement of the hyperdirect pathway between the SMA and STN, resulting in affected cerebellar automatic gait processing.

This study combined different types of overground walking to derive ERP components. When analyzing the ERPs associated with walking, the type of walking movement can affect the resulting ERP waveforms. Figure-eight walking, for example, involves a change in direction and a greater degree of motor planning compared to straight steps [21]. However, the BP associated with turning steps has been shown to have a longer duration and a more complex waveform than that associated with straight steps. These differences in the BP waveform between turning and straight steps may have implications for the interpretation of BP data in studies of walking.

## 5. Conclusions and Future Directions

A limitation of the present study is the relatively small number of participants, particularly for the secondary analysis comparing those participants who definitely exhibited FoG events vs. those who did not, probably, during the experiments (Figures 1(d) and 1(e)). This requires future confirmation with larger groups of participants. This study examined noninvasive cortical biomarkers associated with the decoupling of BG-SMA that causes SMA disruptions. We found that PD patients showed significantly decreased alpha and beta desynchronization in the motor area compared to HC. Furthermore, the late BP slope was also significantly reduced in the PD group compared with the HC, with the definite group showing the largest decrease and consecutively the smallest peak of motor potential. These findings support the theory that the SMA-BG is affected in PD with FoG. Highlighting that this pathway should be an area of focus for pharmacological and nonpharmacological intervention for FoG alleviation, future works will evaluate the effects of dopaminergic medication on the neural biomarkers found and the physical manifestations associated with FoG events. Furthermore, the present findings from persons with definite freezers should be compared in future work to result from persons with PD that do not exhibit the FOG symptom.

## Data Availability

The data used to support the findings of this study are available on request from Meir Plotnik at Meir.PlotnikPeleg@sheba.health.gov.il.

## Supplementary Materials

Supplementary Materials This section provides insights into the methodology and background information of the study. It includes a table presenting participants' demographics, encompassing gender distribution, age, cognitive assessment scores, UPDRS-Part III scores, disease duration, body mass index, and formal education levels. The table incorporates *p* values for significance tests, facilitating comparisons between different groups. In addition, this section outlines the processing of raw EEG data for event-related desynchronization/synchronization (ERD/S) and MRCP analysis, and an accompanying figure illustrates the impact of each preprocessing step on EEG data. Finally, this section introduces the concept of nonparametric permutation testing, employed to address the multiple comparison problem intrinsic to the EEG analysis.
