# Supplementary material for: Supplementary Motor Area Activity Differs in Parkinson's Disease with and without Freezing of Gait
Source: Parkinsons Dis. 2023 Sep 4;2023:5033835. doi: 10.1155/2023/5033835 (PMC10495228; doi:10.1155/2023/5033835)
Supplement: Supplementary Materials — This section provides insights into the methodology and background information of the study. It includes a table presenting participants' demographics, encompassing gender distribution, age, cognitive assessment scores, UPDRS-Part III scores, disease duration, body mass index, and formal education levels. The table incorporates p values for significance tests, facilitating comparisons between different groups. In addition, this section outlines the processing of raw EEG data for event-related desynchronization/synchronization (ERD/S) and MRCP analysis, and an accompanying figure illustrates the impact of each preprocessing step on EEG data. Finally, this section introduces the concept of nonparametric permutation testing, employed to address the multiple comparison problem intrinsic to the EEG analysis. [file 5033835.f1.docx]

# Supplementary materials

# Background material

The Bereitschaftspotential (BP) is a fluctuation that appears over averaged time-locked EEG events, 1-2 seconds before limb movement. The BP is divided into early BP (BP1) and late BP (BP2). Early BP occurs as far back as two seconds before limb movement and is associated with activation at the pre-supplementary motor area (pre-SMA), SMA, and Brodmann area 6 ^1^. The late BP occurs between 400-500 milliseconds before limb movement and is associated with activation of the primary motor cortex (PMC) ^2^. Complementary to the temporal BP, a time-frequency time-locked decomposition yields event-related synchronization and desynchronization. In nominal function, beta synchronization following movement onset antagonizes movement and helps re-establish a postural set; In contrast, desynchronization is related to the preparation of motor responses and motor selection ^3,4^.

Relevant studies have focused on the difference between PD and HC ^5^, or in finding relations between changes in stride length and movement-related cortical potentials (MRCP) in PD with and without FoG ^6^. Recently, researchers showed that the delta and low-beta power band patterns were different between voluntary stops during active gait and stopping due to FoG in PD ^7^.

# Additional material on the Methods

# Participants and Experimental Design

As this is an exploratory study, there is not enough data to establish a valid power analysis. Following similar studies delving into cortical potentials for PD ^8–12^, three experimental groups were investigated: 5 PD patients experiencing FoG during the experiments (herein referred to as having definite FoG), 5 PD patients who had a diagnosis of FoG but who did not exhibit FoG events during the trial (herein referred to as having probable FoG), and 5 age-matched healthy controls (HC). Persons with PD were included if over 50 years of age, diagnosed with idiopathic PD ^13^, and if able to walk 100 m without assistance during the OFF state. Exclusion criteria included significant comorbidities, recent surgeries, or orthopedic impediments. Persons with PD were categorized as suffering from the FoG symptom if they met at least two of the following criteria: (a) the response to the question #2 in the New FoG questionnaire (NFOGQ ^14^), "How frequently do you experience freezing episodes?" was: "more than once a day"; and (b) demonstrated at least two episodes of FoG during short testing for FoG provoking situations, e.g., turns; and (c) were described by the attending neurologist as having a history of FoG. HC were included if over 50 years of age, able to walk without assistance, and had no neurological disease that could affect their gait. Data from these participants were used in part in previous studies ^15,16^. Although all PD participants included in this study met the criteria for FoG classification, not all expressed FoG events during the experimental sessions. Participants' demographics are shown in Table 1. Each PD participant was instructed to arrive on the day of the recording during the OFF dopaminergic state, after at least 12 hours from the last anti parkinsonian medication intake. Full details on data acquisition were previously described ^15,16^. Briefly, each participant performed three gait-related tasks: (1) standing still for one minute, (2) walking with turns, and (3) figure-eight walking.

Table 1.

Subject demographics (mean ± standard deviation).

|  | PD | HC | p-value ^a^ |
| --- | --- | --- | --- |
| Gender (male/female) | 8/2 | 2/3 | 0.606 |
| Age (years) | 67.8 ± 8.4 | 65.2 ± 9.9 | 0.251 |
| Montreal Cognitive Assessment | 22.3 ± 3.9 | 24.8 ± 2.2 | 0.226 |
| UPDRS-Part III | 15 ± 6.8 | N/A | N/A |
| Disease Duration (years) | 11.1 ± 5.0 | N/A | N/A |
| Levodopa equivalent dose (mg) | 910.4 ± 904.9 | N/A | N/A |
| Body Mass Index (kg/m^2)^ | 25.9 ± 4.5 | N/A | N/A |
| Formal Education (years) | 14.2 ± 4.9 | N/A | N/A |

PD: Parkinson's Disease; HC: Healthy Controls; UPDRS: Unified Parkinson's Disease Rating Scale ^17^.
^a^ Randomization test.

# Processing for ERD/S

Raw EEG data was analyzed with EEGLAB ^18^. First, a high-pass filter with cut-off frequency 0.1 Hz was applied to remove low-frequency trends. This was followed by applying the EEGLAB plugin CleanLine ^19^, which utilizes a multi-taper regression technique to remove artifact components of the signal at the line frequency (50 Hz) and its multiples. Then artifact subspace reconstruction (ASR) ^20^ was used to remove flatline and noisy channels, low-frequency drifts, and eye blinks from the data (for ASR performance on EEG movement artifact removal, see e.g. Arad et al.^21^). Following re-referencing to average, ICA was used to estimate cortical potential sources, then visual inspection was used to select and remove artifacts in frequency bands or source locations regarded as noise artifacts.


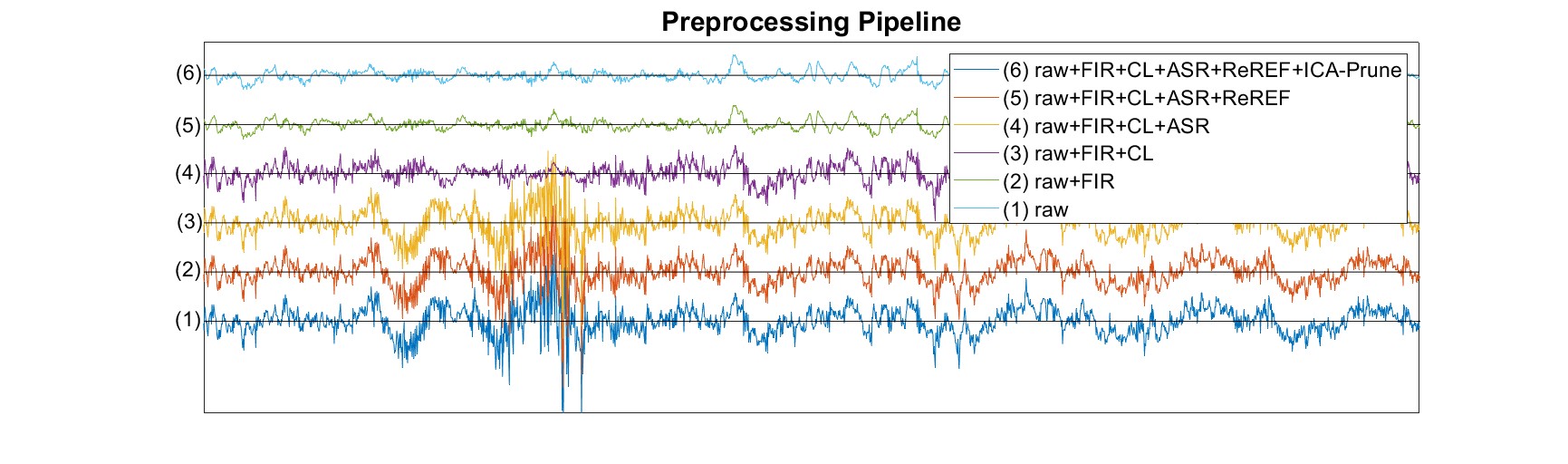


Figure 1. Shows the effects of each preprocessing step on a single EEG channel. These steps focused on removing low-frequency trends, line frequency artifacts, flatline and noisy channels, low-frequency drifts, and eye blinks from the EEG data. On the example above, a strong artifact with characteristic muscle high frequency is removed without affecting the underlying neural activity.

Figure 1. Shows the effect of these processing steps on a segment of data. To estimate the power spectrum for time-frequency analysis, a Morlet wavelet was employed ^22,23^. This wavelet used 0.25 cycles at the lowest frequency which increased linearly to 1.56 cycles at the highest frequency with a window of 17 samples (66.41 ms) covering 24 linearly spaced frequencies from 4 Hz to 50 Hz. After the power spectrum was computed. A baseline from four seconds before to two seconds before gait initiation was selected as a period of no activity ^24^. Each subjects’ baseline was subtracted from their trial and relevant frequency bins were separated for analysis. The relevant frequency bins were alpha at 8, 10, and 12 Hz and beta starting at 14 Hz and going up to 30 Hz by increments of 2 Hz.

# Processing for movement-related cortical potentials (MRCP)

Like processing ERD/S, raw EEG data was input into EEGLAB then a bandpass filter was applied between 0.05 to 10 Hz. This was followed by CleanLine, then artifact subspace reconstruction and re-referencing to average. Then ICA labels were applied, and noisy components or trails were removed. A baseline from two seconds before to one second before gait initiation was selected as a period of no activity. Each subjects’ baseline was subtracted from their trials.

Our methods are limited in terms of baseline corrections. As a feature of time-frequency explorations through EEG, the power change associated with a specific frequency band is measured relative to the power of a baseline period preceding the onset. In the literature, this period is commonly recommended to be recorded several seconds before the onset occurs. Additionally, because of the time required to develop and recover slow alpha rhythms, 10 s between task onsets is recommended. Although the short baseline used in this study (2 s) may negatively affect the quality of the extracted neural patterns (by resulting in a suboptimal correction to the period of inactivity), the success of this trial shows the effectiveness of the results affirms the presence of relevant neural patterns.

# Nonparametric Permutation

Known as randomization or nonparametric permutation testing, is a method for dealing with the multiple comparison problem (MCP) inherent to EEG analysis ^25^. Because EEG data consists of spatio-temporal channel-time pairs, it is very difficult to predict which interactions are significant and analysis requires computing an extremely large number of statistical comparisons that often lead to false pair-wise detections. A permutation distribution is constructed by generating many random draws over equally partitioned groups. To deal with the MCP, the empirical null distribution is constructed using only the maximum t-value for each permuted dataset iteration ^26^. Furthermore, unlike parametric tests, randomization testing does not require the assumption of normal distribution, homogeneous variance, or data independence ^27^.

# Works Cited

1. Lewis SJG, Shine JM. The Next Step: A Common Neural Mechanism for Freezing of Gait. *Neuroscientist*. 2016;22(1):72-82. doi:10.1177/1073858414559101

2. Hallett M. Movement-related cortical potentials. *Electromyography and clinical neurophysiology*. 1994;34(1):5-13.

3. DICK JPR, ROTHWELL JC, DAY BL, CANTELLO R, BURUMA O, GIOUX M, et al. THE BEREITSCHAFTSPOTENTIAL IS ABNORMAL IN PARKINSON’S DISEASE. *Brain*. 1989;112(1):233-244. doi:10.1093/brain/112.1.233

4. Jahanshahi M, Jenkins IH, Brown RG, Marsden CD, Passingham RE, Brooks DJ. Self-initiated versus externally triggered movements. *Brain*. 1995;118(4):913-933. doi:10.1093/brain/118.4.913

5. Vidailhet M, Atchison PR, Stocchi F, Thompson PD, Rothwell JC, Marsden CD. The Bereitschaftspotential preceding stepping in patients with isolated gait ignition failure. *Movement Disorders*. 1995;10(1):18-21. doi:10.1002/mds.870100105

6. Shoushtarian M, Murphy A, Iansek R. Examination of central gait control mechanisms in Parkinson’s disease using movement-related potentials. *Movement Disorders*. 2011;26(13):2347-2353. doi:10.1002/mds.23844

7. Cao Z, John AR, Chen H-T, Martens KE, Georgiades M, Gilat M, et al. *Identification of EEG Dynamics during Freezing of Gait and Voluntary Stopping in Patients with Parkinson’s Disease*.

8. Dick JPR, Cantello R, Buruma O, Gioux M, Benecke R, Day BL, et al. The Bereitschaftspotential, l-DOPA and parkinson’s disease. *Electroencephalography and Clinical Neurophysiology*. 1987;66(3):263-274. doi:10.1016/0013-4694(87)90075-7

9. Georgiev D, Lange F, Seer C, Kopp B, Jahanshahi M. Movement-related potentials in Parkinson’s disease. *Clinical Neurophysiology*. 2016;127(6):2509-2519. doi:10.1016/j.clinph.2016.04.004

10. Shoushtarian M, Murphy A, Iansek R. Examination of central gait control mechanisms in Parkinson’s disease using movement-related potentials. *Movement Disorders*. 2011;26(13):2347-2353. doi:10.1002/mds.23844

11. Stegemöller EL, Allen DP, Simuni T, MacKinnon CD. Motor cortical oscillations are abnormally suppressed during repetitive movement in patients with Parkinson’s disease. *Clinical Neurophysiology*. 2016;127(1):664-674. doi:10.1016/J.CLINPH.2015.05.014

12. Cunnington R, Iansek R, Bradshaw JL. Movement-related potentials in Parkinson’s disease: External cues and attentional strategies. *Movement Disorders*. 1999;14(1):63-68. doi:10.1002/1531-8257(199901)14:1<63::AID-MDS1012>3.0.CO;2-V

13. Hughes A J; Daniel S E; Kilford L; Lees A J. The accuracy of diagnosis of parkinsonian syndromes in a specialist movement disorder service. *Brain*. 2002;125(4):861-870.

14. Nieuwboer A, Rochester L, Herman T, Vandenberghe W, Emil GE, Thomaes T, et al. Reliability of the new freezing of gait questionnaire: Agreement between patients with Parkinson’s disease and their carers. *Gait & Posture*. 2009;30(4):459-463. doi:10.1016/J.GAITPOST.2009.07.108

15. Miron-Shahar Y, Kantelhardt JW, Grinberg A, Hassin-Baer S, Blatt I, Inzelberg R, et al. Excessive phase synchronization in cortical activation during locomotion in persons with Parkinson’s disease. *Parkinsonism Relat Disord*. 2019;65:210-216. doi:10.1016/j.parkreldis.2019.05.030

16. Günther M, Bartsch RP, Miron-Shahar Y, Hassin-Baer S, Inzelberg R, Kurths J, et al. Coupling between leg muscle activation and EEG during normal walking, intentional stops, and freezing of gait in Parkinson’s disease. *Front Physiol*. 2019;10(JUL). doi:10.3389/fphys.2019.00870

17. Fahn, S.; Elton RL. *Recent Developments in Parikinson’s Disease.* Vol 13.; 1986. doi:10.1017/s0317167100036489

18. Delorme A, Makeig S. EEGLAB: an open source toolbox for analysis of single-trial EEG dynamics including independent component analysis. *Journal of Neuroscience Methods*. 2004;134:9-21. doi:10.1016/j.jneumeth.2003.10.009

19. Mullen T. NITRC: CleanLine: Tool/Resource Info. Published 2012. Accessed July 25, 2021. https://www.nitrc.org/projects/cleanline

20. Chang CY, Hsu SH, Pion-Tonachini L, Jung TP. Evaluation of Artifact Subspace Reconstruction for Automatic Artifact Components Removal in Multi-Channel EEG Recordings. *IEEE Transactions on Biomedical Engineering*. 2020;67(4):1114-1121. doi:10.1109/TBME.2019.2930186

21. Arad E, Bartsch RP, Kantelhardt JW, Plotnik M. Performance-based approach for movement artifact removal from electroencephalographic data recorded during locomotion. *PLOS ONE*. 2018;13(5):e0197153. doi:10.1371/JOURNAL.PONE.0197153

22. Pfurtscheller G, Lopes Da Silva FH. Event-related EEG/MEG synchronization and desynchronization: Basic principles. *Clinical Neurophysiology*. 1999;110(11):1842-1857. doi:10.1016/S1388-2457(99)00141-8

23. Shafiul Hasan SM, Siddiquee MR, Bai O. Asynchronous Prediction of Human Gait Intention in a Pseudo Online Paradigm Using Wavelet Transform. *IEEE Transactions on Neural Systems and Rehabilitation Engineering*. 2020;28(7):1623-1635. doi:10.1109/TNSRE.2020.2998778

24. Hasan SMS, Siddiquee MR, Atri R, Ramon R, Sebastian Marquez J, Bai O. Prediction of gait intention from pre-movement EEG signals: a feasibility study. *Journal of NeuroEngineering and Rehabilitation*. 2020;17:50. doi:10.1186/s12984-020-00675-5

25. Maris E, Oostenveld R. Nonparametric statistical testing of EEG- and MEG-data. *Journal of Neuroscience Methods*. 2007;164(1):177-190. doi:10.1016/j.jneumeth.2007.03.024

26. Cardillo G. test-t. GitHub. Published July 7, 2018. Accessed May 6, 2023. https://github.com/dnafinder/testt

27. Mensen A, Khatami R. Advanced EEG analysis using threshold-free cluster-enhancement and non-parametric statistics. *NeuroImage*. 2013;67:111-118. doi:10.1016/j.neuroimage.2012.10.027
